# Photomediated Larvicidal Activity of Pheophorbide a against Cercaria Larvae of* Fasciola gigantica*

**DOI:** 10.1155/2017/5219194

**Published:** 2017-01-03

**Authors:** Divya Jyoti Singh, Vinay Kumar Singh, D. K. Singh

**Affiliations:** Malacology Laboratory, Department of Zoology, D.D.U. Gorakhpur University, Gorakhpur, Uttar Pradesh 273 009, India

## Abstract

Fasciolosis is a parasitic disease caused by* Fasciola gigantica*. The freshwater snail* Lymnaea acuminata* is the intermediate host of* F. gigantica* which cause endemic fasciolosis in the northern part of India. To investigate larvicidal activity of pure and laboratory extracted pheophorbide a (Pa) against cercaria larvae of* F. gigantica*, data were analyzed in different spectra of visible light, sunlight, and laboratory conditions. Photostimulation of chlorophyll derivative pheophorbide a (Pa) caused time and concentration dependent larvicidal activity against cercaria larvae of* F. gigantica*. Larvicidal activity of pure Pa under 650 nm and 400–650 nm (8 h LC_50_ 0.006 mg/10 mL) was more pronounced than extracted Pa under same irradiations (650 nm LC_50_ 0.12 mg/10 mL, 400–650 nm LC_50_ 0.14 mg/10 mL). Lowest toxicity of pure (8 h LC_50_ 0.14 mg/10 mL) and extracted Pa (8 h LC_50_ 1.25 mg/10 mL) was noted under 400 nm. Pa was found to be toxic in laboratory conditions also. The results presented in this paper indicate that pheophorbide a possess potential larvicidal activity against* Fasciola gigantica* larvae in different wavelengths of visible light, sunlight, and laboratory conditions.

## 1. Introduction

Fasciolosis is water borne parasitic zoonoses [1]. Consequences of fasciolosis are the cause of concern in livestock husbandry since long ago [1]. Human fasciolosis incidence has reached to a global estimation of 17 million [2]. Fasciolosis is caused by the trematodes* Fasciola hepatica* and* F. gigantica* [3]. The parasite is transmitted by ingestion of metacercaria of* Fasciola* species on aquatic plants. The infection is most often characterized by fever, pain, eosinophilia, and abdominal inflammation [4]. The intermediate host of liver fluke* F. gigantica* is a hermaphroditic mollusk* Lymnaea acuminata*, inhabiting freshwater ponds and ditches. The liver flukes cause “liver rot” among sheep and cattle which are the definitive hosts; humans are the incidental hosts, by ingesting metacerceria through contaminated water or food [5]. Fasciolosis has the widest geographic spread of any emerging vector-borne zoonotic disease and it is estimated that about 2.4 to 17 million people are infected in more than 51 countries worldwide [2], while 91 million are at risk worldwide [6]. Bovine fasciolosis is very common in eastern region of Uttar Pradesh, India [7]. Although control of snail population below a threshold level is one of the important methods for effective control of fasciolosis, yet snails are one of the important components in the aquatic ecosystem. Release of molluscicides in aquatic ecosystem for snail control also affects the other nontarget organisms. The* Fasciola* larval stages sporocyst, redia, and cercaria are in division phases of* F. gigantica* in the snail body [8]. Now a new approach is considered that if larvae of* Fasciola* will be destroyed in the snail body, the rate of infection can be reduced without killing the snails. The larvicides of plant origin are gaining special importance because their use is economical, safer to nontarget organisms, and culturally more acceptable among livestock keepers [9]. The larvicides of plant origin are gaining special importance in comparison to synthetic counterparts, because they are more effective, cheaper, and safer to nontarget organism and culturally acceptable [10].

Earlier, it has been reported that chlorophyllin and pheophorbide are photodynamically toxic against mosquito larvae and fish parasite in aquatic ecosystem [11–14]. Chlorophyll derivatives like chlorophyllin and pheophorbide have been reported as effective natural photosensitizers against larvae of several insects including flies [15, 16]. Recently D. J. Singh and D. K. Singh [17] reported anthelmintic activity of chlorophyllin against different larval stages of* Fasciola gigantica*. Pheophorbide a (Pa), a main active ingredient of Chinese herbal medicine* Scutellaria barbata* and* Silkworm excreta*, has been proved to be an effective anticancer drug [18, 19]. Recent studies demonstrated that pheophorbide a (Pa) is very photosensitive, and light could significantly activate Pa to deactivate liver cancer cells [19]. Previous studies showed that LED (Light Emitting Diode) could effectively activate Pa and kill colon cancer cells [20]. Photosensitizer is an important factor affecting the successful application of PDT (photodynamic therapy) in the management of malignant tumors. Photoforin, as a first generation photosensitizer, has some drawbacks of prolong skin photosensitivity and weak absorption at long wavelength [21]. Emerging studies have demonstrated that photodynamic action induced by light activated Pa could effectively deactivate liver and colon cancer cells [18, 20]. The objective of the present study is to evaluate the potential efficacy of a newly developed natural plant derived pheophorbide as a photosensitizing agent against the* F. gigantica* larvae.

## 2. Materials and Methods

### 2.1. Animals

Adult* Lymnaea acuminata* each (2.7 ± 0.3 cm in length) were collected locally from Mahesra Lake and low lying submerged areas of Gorakhpur district of Uttar Pradesh, India. Cercariae shedding infected snails were identified according to morphological characteristics (large size, swollen foot, appeared yellowish in colour, slow locomotion, shedding cercaria appeared at the mouth of the snails, changed and shell morphology) as described by Largue et al. [22] and Sunita et al. [8]. The infected snails were allowed to acclimatize for 24 h in laboratory conditions. Each infected snail was dissected in glass Petri dish containing 10 mL of dechlorinated water at 23°C-24°C. After opening the mantle of the snails a large number of redia and cercaria and sporocyst larvae emerged outside the body of snails in Petri dish. In a single dissection of snail about 6357 cercaria larvae can be procured. These larvae survived up to 48 h in laboratory conditions. With the help of micropipette cercaria larvae were separated. The pH of the water was 7.1–7.5, and dissolved oxygen, free carbon dioxide, and bicarbonate alkalinity were 6.3–7.4 mgL^−1^, 5.2–6.4 mgL^−1^, and 103–105 mgL^−1^, respectively. Snail* L. acuminata* and* F. gigantica* were identified by Zoological Survey of India (ZSI), Kolkata [8]. Different wavelengths of visible light were used to study the phototoxicity of pheophorbide.

### 2.2. Test Materials

#### 2.2.1. Pure Pheophorbide a

Pure pheophorbide a (C_35_H_36_N_4_O_5_) was purchased from Sigma-Chemical Co. in the United States.

#### 2.2.2. Preparation of Pheophorbide

**Figure 1:**
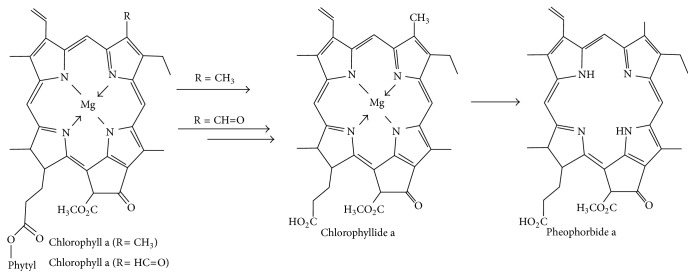


Pheophorbide (Pa) was prepared by the method of Wohllebe et al. [9]. Chlorophyll was isolated from fresh spinach leaves and kept for 2 h in 100% ethanol at 55°C in the incubator. The extract was subsequently filtered with Whatman filter paper and equal volume of petroleum benzene was added. After shaking the mixture in orbital shaking incubator the chlorophyll moved into lipophillic benzene phase. The two phases were separated using a separatory funnel and 1.0 mL HCl was added to 50 mL of benzene phase. On agitation the chlorophyll came into contact with the hydrochloric acid and transformed into water-soluble olive yellow coloured pheophorbide (Figure 1).

### 2.3. Thin Layer Chromatography

Thin layer chromatography (TLC) was performed according to the method of Jaiswal and Singh [23]. Thin layer chromatography was carried out on 20 cm × 20 cm precoated silica gel (Precious Electrochemical industry, Pvt. Ltd. Mumbai, India) using benzene/ethyl acetate (9 : 1, w/v) as the mobile phase. The loading of extracted pheophorbide a with pure pheophorbide was applied on TLC plates with a micropipette. TLC plates were developed with iodine vapour. Copies of chromatogram were made by tracing the plates immediately and *R*_*f*_ value was calculated.

### 2.4. Design of Phototoxicity Response

The photo response experiment was designed by the method of Tripathi et al. [24] and D. J. Singh and D. K. Singh [14]. The protocol of different wavelengths of light production device in the present study is designed by Dr. Ravi Shankar Singh (Associate Professor, Department of Physics, DDU Gorakhpur University, Gorakhpur). Different wavelengths (400–650 nm) of light were separated with the help of interference colour filters. Light intensity was measured against each filter and then output of light was adjusted to get the equal irradiance of 300 W m^−2^ at each band to study the toxicity of pheophorbide a against cercaria larvae.

## 3. Toxicity Determination

### 3.1. In Vitro

In vitro toxicity experiment was performed by the method of D. J. Singh and D. K. Singh [14]. Groups of ten experimental cercaria larvae were added to Petri dishes containing 10 mL dechlorinated tap water. Pure pheophorbide a and extracted pheophorbide a treatment of different concentrations were given directly in the Petri dishes for 4 hours in dark incubation. After the dark incubation period Petri dishes containing treated larvae were irradiated to equal irradiances of 300 W m^−2^ at each wavelength band of visible light for 2 h, 4 h, 6 h, and 8 h. Pure pheophorbide a treatment was made at different concentrations that is 0.01 mg/10 mL, 0.03 mg/10 mL, 0.05 mg/10 mL, and 0.07 mg/10 mL and extracted Pa concentrations 0.1 mg/10 mL, 0.3 mg/10 mL, 0.5 mg/10 mL, and 0.7 mg/10 mL in laboratory, sunlight, and each band of visible light conditions. Each concentration was replicated six times. Mortality data was recorded at each concentration of pheophorbide a treatment in laboratory, sunlight, and equal irradiances of each band of visible light conditions. There were two control groups. In control group I same treatment of pure and extracted Pa was given in darkness without any light exposure and irradiance for same time intervals as in treated groups. In control group II no treatment of Pa was given, while all other conditions were same as with treatment group. Mortality of cercaria larvae was noted under stereomicroscope. Larvae not moving or not showing a vigorous escaping response were defined as dead or dying, respectively, and counted. Concentration-mortality data for each group of larvae were analysed using the probit analysis program, POLO-PC (LeOra Software) [25] to estimate the LC_50_ of pure and extracted Pa and the 95% confidence intervals for these concentrations. The slope of probit lines was also estimated. This program ran chi-square test for goodness-of-fit of the data to the probit model. If the model fits, the calculated value of chi-square is less than the chi-square table value for the appropriate degrees of freedom. If the model does not fit, the LC_50_ value for the particular population may not be reliably estimated and is adjusted with the heterogeneity factor (observed chi-square values divided degrees of freedom). This programme uses heterogeneity factor as a correction factor when the value of Pearson's chi-square statistic is significant at *p* = 0.05. The index of significance for potency estimation (*g*-value) was used to calculate 95% confidence intervals for potency (relative potency is equivalent to tolerance ratio). Parallelism of the probit regression lines implies a constant relative potency at all levels of response. POLO-PC was used to test equality and parallelism of the slope of the probit lines. The regression coefficient analysis between exposure time and different values of LC_50_ was determined by the method of Sokal and Rohlf [26].

## 4. Results

Pure and extracted pheophorbide a exhibited the time and concentration dependent phototoxicity against cercaria larvae of* F. gigantica*. There was a significant regression coefficient between exposure time and LC_50_ of pure and laboratory extracted Pa (Tables 1 and 2). The toxicity of both pure and extracted pheophorbide a was maximum (8 h LC_50_ 0.006 mg/10 mL and 0.12 mg/10 mL, resp.) in 650 nm spectrum band width (red light irradiance) against cercaria larvae of* F. gigantica* (Tables 1 and 2).

The toxicity of pure pheophorbide a in 650 nm band and 400–750 nm band (white light) was almost same (8 h LC_50_ 0.006 and 0.007 mg/10 mL resp.) (Table 1). The maximum phototoxicity of pure pheophorbide a under 650 nm and 400–650 nm band was followed by 590 nm (8 h LC_50_ 0.008 mg/10 mL) and sunlight (8 h LC_50_ 0.008 mg/10 mL) (Table 1). The toxicity of pure pheophorbide a under 570 nm (yellow), 475 nm (blue), and 510 nm (green spectrum) was 8 h LC_50_ 0.02 mg/10 mL, 0.03 mg/10 mL, 0.06 mg/10 mL respectively. Lowest toxicity of pure Pa was noted in 400 nm (violet spectrum) (8 h LC_50_ 0.14 mg/10 mL) and in laboratory conditions (0.07 mg/10 mL) against cercaria larvae (Table 1).

The toxicity of laboratory extracted pheophorbide a in 650 nm band against cercaria larvae was higher (8 h LC_50_ 0.12 mg/10 mL) than 400–650 nm band (white light spectrum) and sunlight exposure (8 h LC_50_ 0.14 mg/10 mL). The toxicity of extracted Pa in 590 nm band (8 h LC_50_ 0.21 mg/10 mL) was followed by 475 nm (8 h LC_50_ 0.24 mg/10 mL), 570 nm (0.27 mg/10 mL), 510 nm (0.41 mg/10 mL), and laboratory (0.85 mg/10 mL) against cercaria larvae (Table 2). Lowest toxicity of extracted Pa was noted in 400 nm spectrum (8 h LC_50_ 1.25 mg/10 mL) (Table 2). Among all the treatments the photolarvicidal activity of pure Pa was higher than extracted Pa for all the conditions against cercaria larvae of* F. gigantica* (Tables 1 and 2). No mortality was noted in both the control groups I and II.

The slope values given in Tables 1 and 2 were steep and separate estimations of LC based on each of the six replicates were found to be within 95% confidence limits of LC_50_. The *t*-ratio is greater than 1.96 and the heterogeneity factor is less than 1. The *g*-value is less than 0.5 at all probability levels (Tables 1 and 2).

The thin layer chromatography analysis demonstrates that the *R*_*f*_ value (0.32) of extracted pheophorbide a was equivalent to pure pheophorbide a (0.32).

## 5. Discussion

It is evident from the above results that the pure and laboratory extracted Pa caused concentration and time dependent larvicidal activity. Toxicity of pure and extracted Pa is higher at 650 nm band against cercaria larvae. It has been reported that chlorophyll derivative chlorophyllin releases more singlet toxic oxygen when exposed to 650 nm and exhibits maximum toxicity against redia and cercaria larvae of* F. gigantica* [14]. Photolarvicidal activity of red spectral band irradiated Pa was followed by white and sunlight irradiance. It has been advocated by various scientists that chlorophyll derivatives like chlorophyllin and pheophorbide show more photomediated control of disease caused by mosquito and insect larvae [12, 13, 16]. The EC_50_ values exposed to pheophorbide for 3 h to a light intensity of 147 W/m^2^ were 8.44 mg/L and 1.05 mg/L against* Culex* and* Chaoborus* mosquito larvae, respectively [11]. LC_50_ values against cercaria larvae of* F. gigantica* is 0.006 mg/10 mL for pure Pa and 0.12 mg/10 mL for extracted Pa exposed to 650 nm spectral band, whereas they are 0.008 mg/10 mL and 0.14 mg/10 mL in sunlight for pure and extracted Pa, respectively. Photodynamic toxicity of pheophorbide a in different spectral band of visible light, white light, and sunlight is most likely due to 4-hour dark incubation which led to significant accumulation of Pa inside the translucent body of cercaria larvae which on photosensitization caused release of free singlet cytotoxic oxygen against cercaria larvae. Wohllebe et al. [9] noted illumination of pheophorbide and chlorophyllin, by visible light start reactions leading to apoptosis and necrosis of the cell in the gut, which ultimately caused death of exposed mosquito larvae. Red light exposed pheophorbide a possess more cercaricidal activity (8 h LC_50_ 0.007 mg/10 mL) than chlorophyllin (8 h LC_50_ 11.99 mg/10 mL) [14]. Wohllebe et al. [9] noted that 24.18 mg/L chlorophyllin and 1.05 mg/L pheophorbide treatment against* Chaoborus* sp. larvae was effective in killing of the larvae.

Toxicity of both extracted and pure Pa in laboratory conditions may be due to accumulation of Pa inside the body of larvae during incubation period and also pheophorbide is reported to be active (less) in darkness [11]. Low toxicity of Pa under violet and green spectral band of visible light is due to its less absorption/production of cytotoxic singlet oxygen species. Although the toxicity of pure Pa was higher than extracted Pa it cannot be neglected that pure Pa is very costly and is not within the reach of the native users for field trials. So we formulated the laboratory extraction of Pa which is tested and harmless in case of ingestion by humans and, in general, by nontranslucent organisms [9].

In conclusion, the pure and extracted pheophorbide a are potent photobiologically active agents even at low concentrations and light intensities. It can be easily formulated into effective and inexpensive photolarvicides. The laboratory studies reported in this work demonstrate the high potential of the photosensitization approach for the control of the larval stages of the* Fasciola* vector. Present study offers promising perspective for the development of a new class of effective and safe control of* Fasciola* infection.

## Figures and Tables

**Table 1:** In vitro toxicity of pure pheophorbide a (Pa) in laboratory, sunlight, and different spectra of visible light conditions against cercaria larvae of *Fasciola gigantica*.

Exposure period	Treatment	LC_50_ (mg/10 mL)	Limits LCL	Limits UCL	Slope value	*t*-ratio	*g*-value	Heterogeneity
2 h	Pa-laboratory	0.53	0.18	10.96	0.98 ± 0.38	2.52	0.60	0.40
	Pa-sunlight	0.05	0.03	0.11	1.06 ± 0.27	3.92	0.24	0.29
	Pa-violet (400 nm)	0.30	0.13	11.41	1.17 ± 0.38	3.87	0.40	0.28
	Pa-blue (475 nm)	0.18	0.16	12.41	1.21 ± 0.36	3.27	0.42	0.22
	Pa-green (510 nm)	0.12	0.08	0.41	1.38 ± 0.34	4.05	0.23	0.17
	Pa-yellow (570 nm)	0.16	0.09	1.19	1.12 ± 0.32	3.50	0.31	0.14
	Pa-orange (590 nm)	0.09	0.05	0.84	1.21 ± 0.32	2.99	0.42	0.16
	Pa-red (650 nm)	0.02	0.01	0.04	0.84 ± 0.25	3.36	0.34	0.14
	Pa-white (400–650 nm)	0.05	0.03	0.11	0.90 ± 0.26	3.38	0.33	0.14

4 h	Pa-laboratory	0.20	0.09	9.37	0.84 ± 0.29	2.86	0.60	0.29
	Pa-sunlight	0.02	0.009	0.032	0.87 ± 0.25	3.69	0.24	0.30
	Pa-violet (400 nm)	0.30	0.13	11.41	1.17 ± 0.38	3.07	0.40	0.28
	Pa-blue (475 nm)	0.18	0.08	1.91	1.06 ± 0.31	3.42	0.42	0.08
	Pa-green (510 nm)	0.10	0.06	0.49	0.98 ± 0.28	3.44	0.23	0.13
	Pa-yellow (570 nm)	0.16	0.09	1.19	1.12 ± 0.32	3.55	0.31	0.14
	Pa-orange (590 nm)	0.04	0.025	0.10	0.76 ± 0.24	2.95	0.42	0.07
	Pa-red (650 nm)	0.01	0.002	0.019	0.84 ± 0.26	4.04	0.34	0.09
	Pa-white (400–650 nm)	0.02	0.016	0.04	0.84 ± 0.25	3.25	0.33	0.15

6 h	Pa-laboratory	0.16	0.07	3.87	0.67 ± 0.27	2.47	0.62	0.10
	Pa-sunlight	0.07	0.002	0.018	0.90 ± 0.26	3.44	0.32	0.40
	Pa-violet (400 nm)	0.14	0.07	1.01	1.00 ± 0.29	3.37	0.33	0.19
	Pa-blue (475 nm)	0.03	0.06	1.21	0.52 ± 0.25	2.06	0.89	0.06
	Pa-green (510 nm)	0.06	0.041	0.43	0.75 ± 0.26	2.83	0.47	0.07
	Pa-yellow (570 nm)	0.06	0.03	0.27	0.78 ± 0.25	2.96	0.43	0.13
	Pa-orange (590 nm)	0.02	0.01	0.03	0.78 ± 0.25	3.21	0.41	0.09
	Pa-red (650 nm)	0.007	0.001	0.012	0.96 ± 0.27	4.89	0.31	0.17
	Pa-white (400–650 nm)	0.01	0.003	0.02	0.78 ± 0.25	3.62	0.41	0.14

8 h	Pa-laboratory	0.07	0.049	0.11	1.04 ± 0.03	3.23	0.38	0.12
	Pa-sunlight	0.008	0.003	0.013	1.26 ± 0.28	5.85	0.19	0.62
	Pa-violet (400 nm)	0.14	0.07	1.01	1.00 ± 0.29	3.37	0.33	0.19
	Pa-blue (475 nm)	0.03	0.06	1.21	0.52 ± 0.25	2.06	0.89	0.06
	Pa-green (510 nm)	0.06	0.041	0.43	0.75 ± 0.26	2.83	0.47	0.07
	Pa-yellow (570 nm)	0.02	0.03	0.27	0.78 ± 0.25	2.96	0.43	0.13
	Pa-orange (590 nm)	0.008	0.01	0.03	0.78 ± 0.25	3.21	0.41	0.09
	Pa-red (650 nm)	0.006	0.001	0.012	0.16 ± 0.26	4.89	0.31	0.17
	Pa-white (400–650nm)	0.007	0.001	0.014	0.86 ± 0.26	4.43	0.26	0.17

Pa: pheophorbide a. Six replicates of ten cercaria larvae were exposed to different concentrations of pure Pa treatment under different light exposure and laboratory conditions. Mortality was recorded every 2 h interval. Concentration given is the final concentration (W/V) in the glass aquarium water. Significant negative regression (*p* < 0.05) was observed between exposure time and LC_50_ of treatments. Ts: testing significant of the regression coefficient, Pa-lab: 15.88^++^, Pa-SL: 16.22^++^, Pa-violet: 7.56^+^, Pa-blue:- 6.10^+^, Pa-green: 8.79^+^, Pa-yellow: 7.64^+^, Pa-orange: 3.80^++^, Pa-red: 12.11^++^, Pa-white: 16.62^++^. +, linear regression between *x* and *y*. ++, nonlinear regression between log *x* and log *y*. LCL: lower confidence limits, UCL: upper confidence limits.

**Table 2:** In vitro toxicity of extracted pheophorbide a in laboratory, sunlight, and different spectra of visible light conditions against cercaria larvae of *Fasciola gigantica*.

Exposure Period	Treatment	LC_50_ (mg/10 mL)	Limits LCL	Limits UCL	Slope Value	*t*-ratio	*g*-value	Heterogeneity
2 h	Pa-laboratory	1.38	0.83	3.30	1.69 ± 0.39	4.27	0.21	0.32
	Pa-sunlight	0.74	0.52	1.53	1.25 ± 0.28	4.33	0.20	0.28
	Pa-violet (400 nm)	1.53	0.94	5.64	1.64 ± 0.41	3.95	0.24	0.18
	Pa-blue (475 nm)	0.84	0.49	5.54	0.81 ± 0.27	3.00	0.42	0.08
	Pa-green (510 nm)	1.19	0.79	3.05	1.61 ± 0.37	4.35	0.20	0.34
	Pa-yellow (570 nm)	1.19	0.79	3.05	1.61 ± 0.37	4.35	0.20	0.34
	Pa-orange (590 nm)	0.56	0.39	1.09	1.01 ± 0.27	4.04	0.23	0.16
	Pa-red (650 nm)	0.34	0.24	0.49	1.17 ± 0.26	4.40	0.19	0.45
	Pa-white (400–650 nm)	0.43	0.34	0.58	1.61 ± 0.28	5.68	0.12	0.18

4 h	Pa-laboratory	1.36	0.84	5.44	1.31 ± 0.33	3.88	0.25	0.18
	Pa-sunlight	0.42	0.31	0.64	1.22 ± 0.27	4.53	0.18	0.27
	Pa-violet (400 nm)	1.36	0.83	5.13	1.30 ± 0.33	3.93	0.24	0.18
	Pa-blue (475 nm)	0.31	0.25	0.55	1.09 ± 0.26	4.11	0.22	0.11
	Pa-green (510 nm)	0.90	0.63	1.90	1.44 ± 0.31	4.55	0.18	0.14
	Pa-yellow (570 nm)	0.58	0.40	1.25	1.03 ± 0.27	3.80	0.26	0.10
	Pa-orange (590 nm)	0.40	0.30	0.59	1.27 ± 0.27	4.70	0.17	0.35
	Pa-red (650 nm)	0.22	0.24	0.30	1.23 ± 0.26	4.67	0.17	0.34
	Pa-white (400–650 nm)	0.30	0.21	0.40	1.35 ± 0.26	5.01	0.15	0.10

6 h	Pa-laboratory	1.26	0.77	4.87	1.21 ± 0.31	3.85	0.25	0.02
	Pa-sunlight	0.24	0.14	0.33	1.13 ± 0.26	4.31	0.20	0.11
	Pa-violet (400 nm)	1.33	0.79	5.63	1.97 ± 0.31	3.78	0.26	0.05
	Pa-blue (475 nm)	0.31	0.20	0.47	1.00 ± 0.26	3.83	0.26	0.11
	Pa-green (510 nm)	0.63	0.47	1.07	1.40 ± 0.29	4.80	0.16	0.20
	Pa-yellow (570 nm)	0.37	0.27	0.53	1.27 ± 0.27	4.70	0.17	0.18
	Pa-orange (590 nm)	0.32	0.23	0.44	1.27 ± 0.26	4.74	0.17	0.45
	Pa-red (650 nm)	0.17	0.11	0.23	1.41 ± 0.26	5.25	0.13	0.29
	Pa-white (400–650 nm)	0.21	0.15	0.27	1.51 ± 0.27	5.61	0.12	0.06

8 h	Pa-laboratory	0.85	0.52	3.9	0.89 ± 0.27	3.26	0.36	0.07
	Pa-sunlight	0.14	0.07	0.19	1.33 ± 0.26	4.97	0.15	0.17
	Pa-violet (400 nm)	1.25	0.76	4.89	1.20 ± 0.31	3.82	0.26	0.03
	Pa-blue (475 nm)	0.24	0.13	0.35	0.96 ± 0.26	3.71	0.27	0.10
	Pa-green (510 nm)	0.41	0.29	0.63	1.16 ± 0.26	4.34	0.20	0.24
	Pa-yellow (570 nm)	0.27	0.19	0.37	1.28 ± 0.26	4.83	0.16	0.12
	Pa-orange (590 nm)	0.21	0.14	0.28	1.32 ± 0.26	4.96	0.19	0.45
	Pa-red (650 nm)	0.12	0.05	0.18	1.20 ± 0.26	4.53	0.19	0.28
	Pa-white (400–650 nm)	0.14	0.09	0.19	1.51 ± 0.27	5.53	0.12	0.29

Pa: pheophorbide a. Six replicates of ten cercaria larvae were exposed to different concentrations of laboratory extracted Pa treatment under different light exposure and laboratory conditions. Mortality was recorded every 2 h interval. Concentration given is the final concentration (W/V) in the glass aquarium water. Significant negative regression (*p* < 0.05) was observed between exposure time and LC_50_ of treatments. Ts: testing significance of the regression coefficient, Pa-lab: 3.70^++^, Pa-SL: 6.73^+^, Pa-violet: 20.9^+^, Pa-blue: 11.42^++^, Pa-green: 9.98^+^, Pa-yellow: 2.63^++^, Pa-orange: 9.47^+^, Pa-red: 8.14^+^, Pa-white:- 8.74^++^. +, linear regression between *x* and *y*. ++, nonlinear regression between log *x* and log *y*. LCL: lower confidence limits, UCL: upper confidence limits.
